# A Single Nucleotide Polymorphism in cBIM Is Associated with a Slower Achievement of Major Molecular Response in Chronic Myeloid Leukaemia Treated with Imatinib

**DOI:** 10.1371/journal.pone.0078582

**Published:** 2013-11-05

**Authors:** Vanessa Augis, Kelly Airiau, Marina Josselin, Béatrice Turcq, François-Xavier Mahon, Francis Belloc

**Affiliations:** 1 Université Bordeaux Segalen and INSERM U1035 Bordeaux, Bordeaux, France; 2 Laboratoire d’Hématologie, Centre Hospitalier Universitaire de Bordeaux, Bordeaux, France; 3 Service des maladies du Sang, Centre Hospitalier Universitaire de Bordeaux, Bordeaux, France; Institut national de la santé et de la recherche médicale (INSERM), France

## Abstract

**Purpose:**

BIM is essential for the response to tyrosine-kinase inhibitors (TKI) in chronic myeloid leukaemia (CML) patients. Recently, a deletion polymorphism in intron 2 of the BIM gene was demonstrated to confer an intrinsic TKI resistance in Asian patients. The present study aimed at identifying mutations in the BIM sequence that could lead to imatinib resistance independently of BCR-ABL mutations.

**Experimental Design:**

BIM coding sequence analysis was performed in 72 imatinib-treated CML patients from a French population of our centre and in 29 healthy controls (reference population) as a case-control study. Real-time quantitative PCR (RT qPCR) was performed to assess Bim expression in our reference population.

**Results:**

No mutation with amino-acid change was found in the BIM coding sequence. However, we observed a silent single nucleotide polymorphism (SNP) c465C>T (rs724710). A strong statistical link was found between the presence of the T allele and the high Sokal risk group (p = 0.0065). T allele frequency was higher in non responsive patients than in the reference population (p = 0.0049). Similarly, this T allele was associated with the mutation frequency on the tyrosine kinase domain of BCR-ABL (p<0.001) and the presence of the T allele significantly lengthened the time to achieve a major molecular response (MMR). Finally, the presence of the T allele was related to a decreased basal expression of the Bim mRNA in the circulating mononuclear cells of healthy controls.

**Conclusion:**

These results suggest that the analysis of the c465C>T SNP of BIM could be useful for predicting the outcome of imatinib-treated CML patients.

## Introduction

Chronic myeloid leukaemia (CML) is a myeloproliferative disorder characterized by the t(9;22) translocation leading to the fusion transcript BCR-ABL. This transcript encodes a deregulated chimeric tyrosine kinase. Small-molecules inhibiting Bcr-Abl tyrosine-kinase activity (TKI) such as imatinib mesylate have fundamentally improved the treatment of CML ; however, some patients do not undergo an optimal response to imatinib [1]. In chronic phase, this response failure can be explained in about 25% of cases by the growth of CML cells that exhibit point mutations in the BCR-ABL kinase domain [2], [3]. The anti-leukemic mechanism of imatinib is to selectively inhibit the growth of CML cells and to induce apoptosis [4]. RNA interference experiments have demonstrated that the pro-apoptotic protein BIM (a protein belonging to the Bcl-2 family proteins) is essential to this apoptotic signal [5]–[7]. Thus, mutations in the BIM sequence could lead to imatinib resistance beside the appearance of the BCR-ABL kinase domain mutation. Recently, a deletion polymorphism in intron 2 of the BIM gene was demonstrated to confer a TKI resistance in Asian patients [8].

In the current work, we performed BIM coding sequence analysis for imatinib responder and non responder CML patients. We did not find any mutation with amino-acid change in the coding sequence in any of the 72 patients analyzed. A single nucleotide polymorphism (SNP) located in exon 5 of the BIM gene was observed in our French population. The presence of the T allele in the c465C>T SNP was significantly associated with a longer delay to achieve a major molecular response (MMR) leading to more frequent mutations in the kinase domain of BCR-ABL and to TKI resistance.

## Materials and Methods

### Ethics Statement

Written informed consent was obtained in accordance with the Declaration of Helsinki from all patients and from parents or guardians on behalf of children who participated in this study, which was performed parallel to the molecular evaluation.

The study was approved by the local Ethics Committee : Comité Consultatif de Protection des Personnes dans la Recherche Biomédicale (CCPRB) de Bordeaux at the University of Bordeaux.

### Patients and healthy controls

All CML samples were supplied by the tumor bank of the Haut Lévêque Hospital (Pessac, Bordeaux, France) and obtained from patients who provided an informed consent. Among them, 56 samples from CML patients were presented to the Bordeaux Hospital between January 2005 and September 2008 for BCR-ABL tyrosine-kinase domain (TKD) sequencing. All patients with excess samples (46) were sequenced for the BIM gene. A randomly chosen group of 26 CML patients with optimal response to imatinib was also sequenced for the BIM gene afterwards. The BCR-ABL TKD could not be sequenced for these patients due to the low level of BCR-ABL mRNA available at the time of the analysis. Median age was 60 years (range 5 to 92), 30 patients were females and 42 males (*sex ratio*: 1.4). The disease progressed at least once for 25 patients in accelerated phase (AP, 21 patients) or in blastic phase (BP, 4 patients). All patients were treated with 400 mg imatinib per day at the time of their inclusion in the study.

Optimal response, suboptimal response, and failure to imatinib first line therapy were defined according to the European Leukaemia net recommendations [1]. We designated 2 groups of patients: the “non responders” *i.e*. patients with failure or suboptimal response and the “responders” *i.e.* patients with optimal response.

Additional cytogenetic anomalies were found in the progressive phases of the disease since there are criteria for acceleration. From the 46 patients analysed for a *BCR-ABL* mutation, 13 exhibited *BCR-ABL* mutations. They included the E355G (n = 3), T315I (n = 3), F317L (n = 3), M351L (n = 1), V299L (n = 2), H396R and F359V (n = 2) mutations. Two patients harboured a double mutation.

The follow-up of BCR-ABL expression was available for 64 patients and 39 obtained a MMR in less than 18 months and were considered as responders. Patients’ characteristics are summarized in Table 1.

**Table 1:** Patients characteristics.

	Total (n = 72)	BIM 465 C/C (n = 32)	BIM 465 C/T (n = 34)	BIM 465 T/T (n = 6)
Age in years				
Median (range)	60 (5–92)	60 (14–79)	60 (5–92)	58 (35–71)
Sex				
Sex ratio	1.40	1.75	1.27	0.5
Sokal risk score^a^				
Median	0.90	0.90	0.89	0.88
Low (n)	19	10	7	2
Intermediate (n)	15	8	6	1
High (n)	14	3	11	0
MMR b(n/available)				
> 18 months (n)	25/64	7	17	1
≤ b 18 months (n)	39/64	22	13	4
Additional cytogenetic anomalies^c^ (n)	17	8	7	2
Progressive phases^d^ (n)	25	9	13	3
TKD mutations (n/sequenced)	13/46	2/19	9/23	2/4

a The Sokal score was known for 48 patients/72.

b The number of patients achieving or not a major molecular response (MMR) is shown. MMR was defined as a BCR-ABL/ABL ratio ≤ 0.1% on the international scale.

c The number of patients for whom one (or several) additional cytogenetic anomaly(ies) was (were) detected in Ph+ mitosis.

d The number of patients who underwent either the acceleration or blastic phase at least once during the study.

A cohort of 30 voluntary healthy controls (considered as a reference group) recruited among the hospital staff was also analysed for BIM sequencing (29 available) and for BIM expression by RT-qPCR.

### Amplification and sequencing

Whole leukocyte fraction was obtained from buffy coat followed by red cell lysis (using ammonium chloride). Total RNA extraction was conducted using the Trizol method (Invitrogen). Complementary DNA was generated using random primers and AMV reverse transcriptase (Roche) according to the manufacturer’s instructions.

- Molecular response to imatinib was assessed by quantifying the BCR-ABL transcript level using reverse-transcriptase quantitative polymerase chain reaction (RT qPCR), according to the recommendations proposed for result harmonization [9]. MMR was defined as a BCR-ABL/ABL ratio ≤ 0.1% on the international scale [1].

- BCR-ABL TKD sequencing was performed as previously described [10].

- Complete *BIM* coding sequence was amplified using the Expand long template PCR system kit (Roche) and the following primers: 5′-TTCCCCCAAATGTCTGACTC-3′ (forward) and 5′-GTGCTGGGTCTTGTTGGTTT-3′ (reverse). BIM cDNA sequencing was not possible after this amplification (pink arrows, Figure S1b), due to the presence of several isoforms that do not share the same sequence (See Figure S1a). Thus, the sequencing strategy (Figure S1b) focused on sequencing BIMEL, [11] the longest isoform generated by alternative splicing that contains coding information of the whole coding sequence. For this, primers were designed (yellow arrows) to anneal to exon 3 that is excised during alternative splicing of the other isoforms. The Big Dye terminator V 1.1 cycle sequencing kit (Applied Biosystems) was used for sequence reaction. Sequence primers were: 5′-CAGAGCCACAAGGTAATCCTG-3′ (forward) and 5′-TACCCACTGGAGGATCGAGA-3′ (reverse).

- Capillary electrophoresis was conducted using the 3130 Genetic Analyser (Applied Biosystems) and the sequences obtained were analysed using the DNA sequencing analysis software (Applied Biosystems). SNPs were detected with the Seqscape software (Applied Biosystems) and visually controlled.

### Analysis of BIM expression by RT-qPCR

cDNA was synthesized from 1 µg of total RNA with random hexamers in a final volume of 20 µL of RT reaction buffer (Roche). Quantitative RT-PCR was carried out in 96-well ABgene plates using the Mx3005P system (Stratagene) with the SYBR Green Master Mix reaction (Stratagene). All reactions were performed in a total volume of 18 µL and contained 2 µL of cDNA diluted 10 folds and 6.25 µM of each primer set. Triplicate samples were analysed for each data point. Negative controls without reverse transcriptase added were performed. The primer sequence information were: BIM forward primer 5′- GAAGGCAATCACGGAGGTGA-3′ and reverse primer 5′- AGGACTTGGGGTTTGTGTTG -3′, *ß-actin* forward primer 5′-AGCATCGGGTGATGTTCATT-3′ and reverse primer 5′-ATTACAAGCATGCGTCACCA-3′. Thermal cycling was performed at 95°C for 3 min followed by 40 cycles comprising each a denaturation step at 95°C for 15 s and an annealing/extension step at 60°C for 22 s. Amplification of the appropriate product was verified by continuous monitoring fluorescence through the dissociation temperature of the PCR product at a temperature transition rate of 0.1°C/s to generate a melting curve. PCR products of ß-actin and *BIM* were cloned into the pCR®2.1-Topo vector using the TOPO TA Cloning® kit (Invitrogen) and dilutions of these plasmids were used to generate a calibration curve. Triplicate assays of serial dilutions of these plasmids were included in each experiment to produce the calibration curves. *BIM* levels were expressed as the ratio of the number of transcripts to the number of *ß-actin* control transcripts in each sample.

### Cell lines

The K562 cell line, obtained from European collection of cell cultures (ECACC), comes from a CML patient and expresses the non mutated BCR-ABL fusion gene. Cell lines were maintained in RPMI 1640 medium (Biowest) supplemented with foetal calf serum (10% v/v), 10 mM Hepes, 100 units/ml penicillin, 50 µg/ml streptomycin in a humidified atmosphere containing 5% v/v CO_2_ at 37°C. Exponentially growing cells were used in all experiments.

The K562 imatinib-resistant cell line [12] was maintained in the presence of 1 µM imatinib in culture medium. It has no BCR-ABL mutation. BIM sequencing was conducted as described for the patients.

### Statistical analysis

To ensure statistical analysis and to avoid false associations, the Hardy-Weinberg exact equilibrium test and the analysis of SNP association with response status were performed with the online SNPstats software [13]. This web tool analyses associations by logistic regression and interactions by a global test and by a test for the integration in the linear trend of the nested variable [14]. Statistical analysis for Fisher’s exact, Chi square, Z, Mann-Whitney and Kaplan-Mayer tests were conducted using the MedCalc statistical analysis software. A p value <0.05 was considered as significant.

## Results

### Evidence for a polymorphism in the coding region of BIM mRNA

After cDNA amplification of BIM, we observed several isoform combinations (See Figure S1a). In K562 cell lines (Lane 1 and 2), the 3 main isoforms BIM_EL_, BIM_L_ and BIM_S_ were present among others. K562 imatinib-resistant and -sensitive cells presented the same BIM isoforms. PCR products from patients in the different phases of the disease (Lane 3 to 5) also showed the 3 main isoforms but harboured more isoforms than the cell lines.

**Figure 1:**
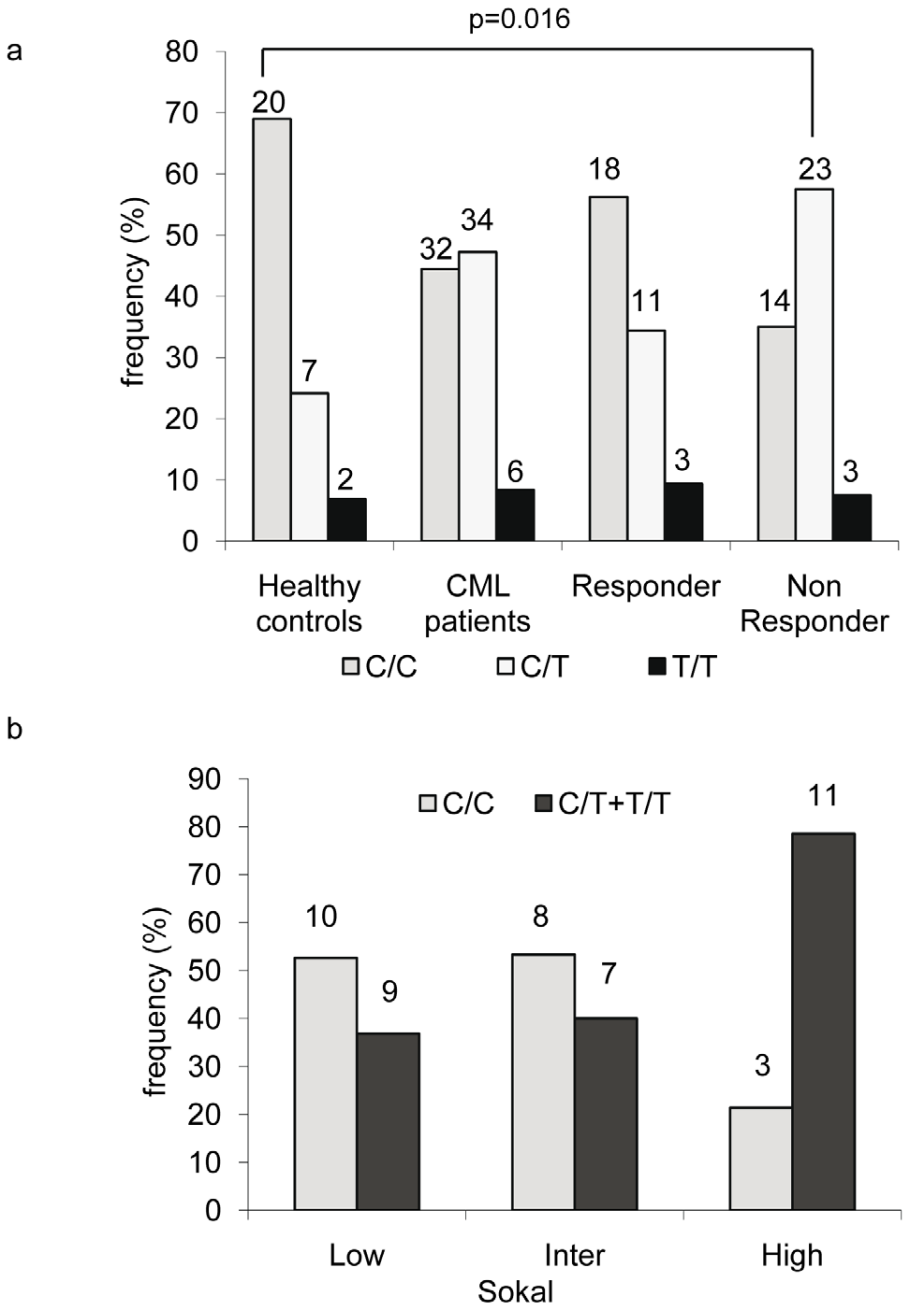
Relation between the c465C>T genotypes and the Sokal risk score or imatinib response. **a:** BIM_EL_ coding region was sequenced in the cDNA from 72 CML patients and 29 healthy donors. The c465C>T genotype frequency (in %) was plotted for the healthy controls and the whole patient cohort. In this cohort, 32 were responders (optimal response) while 40 “non responders” exhibited sub-optimal or failure to first line imatinib treatment. Each bar represents the frequency percentage of the corresponding genotype in each group. **b:** cDNAs from 48 patients were sequenced for the BIM_EL_ coding region and the frequency of C/C or C/T+T/T genotypes was plotted for risk subcategories according to the Sokal score: <0.8 characterizes the low risk group, >1.2 the high risk group and 0.8<–<1.2 the intermediate group. The bars represent the frequency (in percentage) of the C/C (light grey) and C/T+T/T (dark grey) genotype. The number of cases was indicated above each bar?

We did not find any mutation that led to amino-acid change in the coding sequence of BIM from any of the 72 patients sequenced. However, a silent polymorphism located in exon 5 was identified. This synonymous SNP changes the codon from ATC (Ile) to ATT (Ile) (See figure S1c). The polymorphism c465C>T is located at the nucleotide position 465 (from the AUG of the starting codon in exon 2) and is indexed in the NCBI dbSNP database (rs724710) [15], [16]. We observed 3 genotypes (electrophoregrams showing examples of the sequences obtained are presented in figure S1c): homozygous C/C genotype, base C being the ancestral allele (upper chart), homozygous T/T genotype (middle chart) and heterozygous C/T genotype (lower chart). The overall frequencies for BIM 465 C/C, C/T and T/T genotypes found in CML patients (n = 72) were 44%, 47% and 8%, respectively and were significantly different than those of the local healthy control reference group (OR, 2.78; CI, 1.11–6.93; p = 0.024), for which the respective frequencies were 69, 23 and 8% (Figure 1a). The genotype frequency distribution was in the Hardy-Weinberg equilibrium (p = 0.59 for the patients and p = 0.24 for the reference series). The minor allele frequency was greater than 1%. Thereafter, we analysed the impact of this SNP on CML patients in relation with the response to the treatment and progression of the disease.

### The c465C>T SNP is linked to the response to imatinib

The patients were classified as responder or non responder on the basis of their ability to achieve a MMR at 18 months of treatment with imatinib (Table 1). The two groups did not differ in respect of age or sex ratio. However, the non-responder group presented more frequently a high Sokal risk score (p = 0.006) at diagnosis, additional cytogenetic anomalies (p = 0.002) and/or a progression (p = 0.003) during the follow-up than the responder group and included all the mutations in the BCR-ABL TDK.

The presence of the T allele was more frequent in the high Sokal risk group than in the low and intermediate risk groups (Figure 1b, OR = 0.197, 95% CI 0.05 to 0.85, p = 0.03 using the Fisher’s exact test). Moreover, interaction analysis of the BIM 465 SNP with the Sokal risk groups using the SNPstats web tool showed a p value of 0.0065. The test for interaction in the trend for the Sokal group within SNP gave a value p = 0.037 and reciprocally, SNP within the Sokal group gave a value p = 0.0022, confirming the strong interaction between both covariates. Interestingly, the 11 patients with a high risk score associated with a C/T genotype were found in suboptimal or failure response to imatinib.

**Figure 2:**
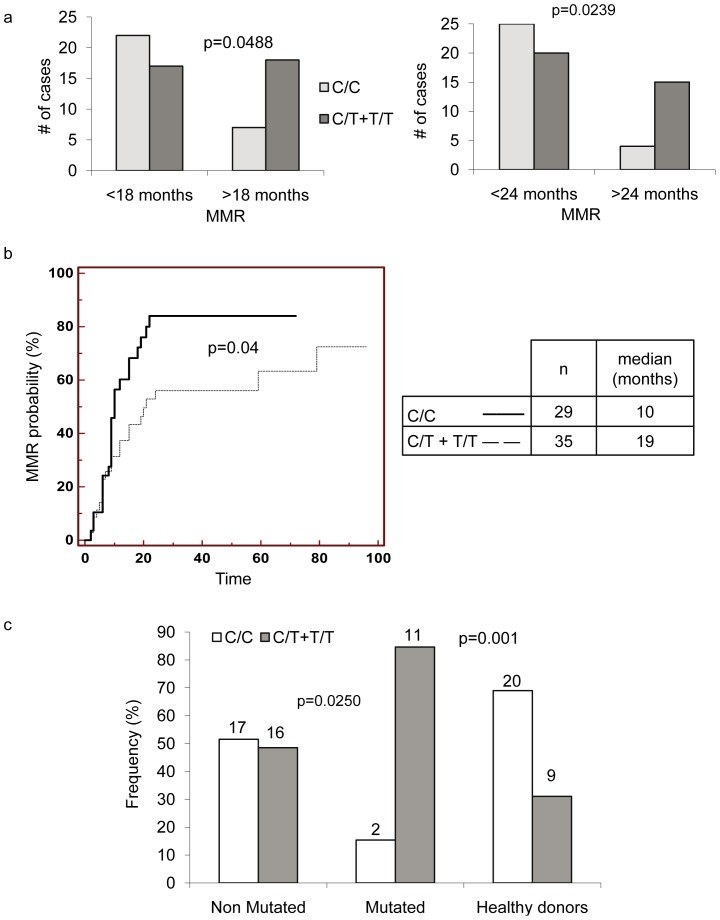
Relation between the C/C or T/T plus C/T BIM genotypes and the achievement of MMR. **a**: cDNAs from 64 patients followed in the laboratory for their Bcr-Abl expression were sequenced for the BIM_EL_ coding region. The number of patients achieving a MMR before or after 18 months (left panel) or 24 months (right panel) was plotted as a function of the c465C>T genotype: C/C (white bars) or C/T plus T/T (grey bars). p indicates significance with the chi-square test. **b**: Kaplan-Meier analysis of the achievement of a MMR. The figure represents the probability to achieve a MMR (in %) as a function of the treatment duration (in months) for patients with the C/C genotype (continuous line) or patients with the C/T or T/T genotype (dotted line). **c**: cDNAs from 37 resistant patients were sequenced for both the BCR-ABL tyrosine kinase domain (TKD) and BIM_EL_ coding region. The frequency of patients with non mutated or mutated TKD was plotted as a function of the c465C>T genotype. The numbers above the bars indicate the sample size. The p value was calculated using the Chi-square test.

When the frequencies of the C/C, C/T and T/T genotypes in the responsive and resistant groups were compared to those of the local reference group (healthy controls) (Figure 1a), no significant difference was evidenced between the responder and reference groups. However, the distribution of the genotypes in the non responder group differed from that in the reference group (p =  0.016 using the chi square test) with an increased frequency of the T allele. This result was confirmed by the analysis of the non responder group compared to the local reference group using the SNPstats web tool showing an association of the genotype with the non responder phenotype in the dominant mode (OR, 4.13; CI, 1.49–11.45; p = 0.0049). We compared the c465C>T SNP genotype frequency in the patients obtaining a MMR in less than 18 months with those necessitating more time. Figure 2a, left panel, shows that the T allele was more frequently found in patients who do not reach a MMR in 18 months, i.e. patients in suboptimal response (chi square 3.88, p = 0.0488). The significance was better for a cut-off of 24 months to achieve a MMR (figure 2a, right panel, chi square 5.101, p = 0.0239). Moreover, a Kaplan-Meier analysis of the MMR frequency as a function of the treatment duration (figure 2b) confirmed that the presence of the T allele lengthened the time for achieving a MMR (p = 0.0407).

Because more than 30% (in our series) of non responder patients presented a mutation in the TKD of *ABL*, it was interesting to check if the c465C>T SNP genotype could be another predictive factor of resistance independently of the mutational status of the *BCR-ABL* gene. When the frequency of the genotype was plotted as a function of the mutational status in non responder patients, we observed (Figure 2c) that the T allele was more frequent in mutated than in non mutated patients (p = 0.0250) or in reference group (p = 0.0013), while the frequency in non mutated patients did not differ from the local reference group (p = 0.16). Thus, we performed an association analysis of SNP with the mutational status using SNPstats and found a strong association (OR, 12.22; CI, 2.23–66.88; p<0.001) in a dominant mode. Conversely, no association was found between the SNP genotype and the frequency of additional chromosomal alterations.

**Figure 3:**
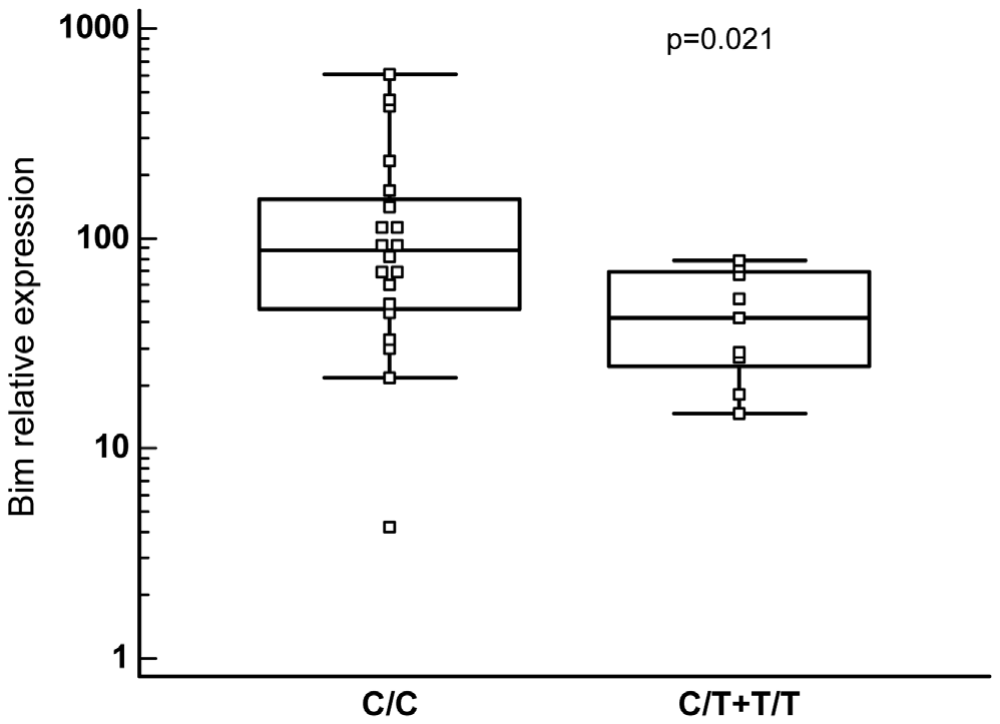
Relation between the C/C or C/T plus T/T BIM genotypes and the basal expression of Bim. The cDNA from mononuclear cells of 29 healthy controls was analysed for BIM mRNA expression by quantitative RT-PCR and sequencing of the BIM coding region. The BIM expression (relative to β actin) was plotted as a function of the c465C>T genotype comparing the C/C to the C/T and T/T genotypes. The p value was calculated using the Mann-Whitney test.

We then wondered if the c465C>T SNP genotype could influence the expression level of Bim. If this is the case, it is likely independent of the Bcr-Abl expression and thus occurs in non leukemic cells as well. Bim_EL_ is the most regulated isoform during imatinib treatment [7] and thus, we analysed the expression level of its mRNA as a function of the c465C>T genotypes in normal mononuclear cells isolated from the blood of 29 healthy controls. It was found (Figure 3) that the T allele was associated with a reduced expression of this mRNA (p = 0.0209). Moreover, only mononuclear cells expressing the C/C genotype were able to produce a Bim/β actin ratio greater than 100 (8/20). This suggests that cells expressing the C/C genotype had a better ability to respond to demand peaks in Bim than the cells expressing the C/T or T/T genotypes.

## Discussion

In preliminary experiments, we sequenced the *BCR-ABL* fusion gene in a series of patients treated in the haematology centres of Bordeaux. Amongst them, 29% only of non responder patients in chronic phase exhibited a point mutation in the *BCR-ABL* gene. Thus, mutations in the *BCR-ABL* TKD were not involved in inadequate responses for most cases. The pro-apoptotic protein BIM plays a central role in apoptosis of *BCR-ABL* cells in response to tyrosine kinase inhibitors (TKI) [5]–[7]. It has been proposed as a tumor suppressor in mouse B-cell leukemia and in human B-cell lymphomas [17], [18], and its dysfunction or altered expression could lead to TKI resistance in CML patients. However, we did not detect any point mutation or any other sequence anomaly susceptible to change the BIM amino-acid sequence in our local patient series. This does not exclude the occurrence of some qualitative acquired alterations in the BIM sequence but could only reflect the sensitivity limit of the method. Actually, the median relative expression of *BCR-ABL* was only 0.01% (range 0–65) in the responder group and 7.09% (range 0–100) in the non responder group at the time of *BIM* sequencing. If a *BIM* mutation occurred in *BCR-ABL* expressing cells, its detection would be impeded by the abundance of normal sequences in the non leukemic cells which also expressed *BIM*. In 13 samples, however, the relative *BCR-ABL* expression was higher than 20% and in these conditions, a mutation in *BIM* would have been detected if present but none of these patients had a mutation in the coding sequence. Consequently, on the basis of our results, the hypothesis that a qualitative deficiency of BIM provides an advantage to the cells during TKI treatment was not verified.

Nevertheless, our study showed that an intragenic SNP: c465C>T in the *BIM* gene generates three genotypes. Despite the relatively small sample size, the strength of this case-control study ensues from the fact that both patient and control participation was restricted to individuals residing in the Aquitaine region in France. The impact of this silent polymorphism c465C>T (rs724710) has never been studied before. The genotype distribution in CML patients differed from that found in a random population and this discrepancy was essentially due to the non responder patient group. The T allele was found here significantly associated with a higher risk of disease at diagnosis and, furthermore, with detrimental factors such as the appearance of mutations in the BCR-ABL TDK. The c465C>T SNP is located in the region coding for the structured and active part of the BIM protein (BH3). In the TT genotype, only the ATT (Ile) codon is used. This codon is supposed to be rare as described for the *MDR1* gene [19] (RSCU values change from 20.9 for ATC to 15.8 for ATT) although its impact was evaluated in haplotypes of rare codons. Therefore, c465C>T SNP may have an impact on the speed of the BIM translation and consequently on the BIM folding and activity as previously suggested for the *MDR1* and *DRD2* genes [19]–[22]. Additionally, although located in the coding sequence, this polymorphism could be in linkage disequilibrium with other point mutations in a non-coding part of the gene. For example, other SNPs linked to the c465C>T SNP could be located in the 3′ untranslated terminal region (UTR) of the *BIM* gene and some of them could interfere with the miRNA binding [23], [24]. Other SNPs in intronic and UTR regions of the *BIM* gene were previously associated with a risk of developing non-Hodgkin lymphoma and some of them were in linkage disequilibrium with the c465C>T SNP [25]. Similarly, SNPs in the promoter region could quantitatively reduce its expression. Twenty six SNPs located in the *BIM* promoter are currently indexed [26]. Unfortunately, the genomic DNA was not available for any of the CML patients studied here and thus the promoter region of the *BIM* gene could not be investigated in this series. However, this last hypothesis is supported by the finding here that the T allele in 465 was associated with a reduced basal expression of the *BIM* mRNA in normal blood cells. It is known [7] that leukemic cells deprived in BIM content are poor responder to TKI and it is possible that cells with decreased levels of BIM exhibit a slower response to these TKI. On an other hand, in patients, time response to TKI higher than 12 months are considered as favouring the occurrence of resistance and thus the detection of TKD mutations in BCR-ABL. Thus, it is possible that C/T and T/T genotypes, by lowering the BIM expression induced a lengthening of the response thus facilitating the occurrence and the selection of mutated clones in the leukemic stem cells population. Alternatively, these haplotypes could be related to polymorphisms in spliced elements like introns or in consensus splicing sequences leading to an accumulation of inactive or less active isoforms. Several inactive isoforms have previously been described for BIM [27], [28] and were found in the patients and cell lines studied (Data not shown and supporting figure S1a). Thus, the quantitative analysis of mRNA for each of these isoforms could provide information on cellular ratios. Moreover, such a polymorphism leading to an inactive isoform was recently evidenced in Asian patients and was related to a TKI resistance [8]. However, this polymorphism common in East Asian individuals (5 to 12%) was not found in the European population. The SNP described here concerns far more people, namely 30% in our French population, and could be of great importance in the response to treatment.

Nevertheless, we report here a relationship between the *BIM* genotype and response of CML during imatinib treatment. SNPs are inherited genomic point mutations. This leads us to consider that some CML patients could have a genomic predisposition to poorly respond to treatment and thus could benefit from a personalized biological monitoring and treatment adaptation particularly associating BH3 mimetics to TKI as previously suggested [8], [29] for compensating the Bim deficiency.

## Supporting Information

Figure S1 **BIM_EL_ cDNA amplification and sequencing.** ***a:*** *Agarose gel electrophoresis of PCR products after BIM cDNA amplification*. PCR products after BIM cDNA amplification were analysed by agarose gel electrophoresis. cDNAs were obtained from: K562 imatinib-sensitive cell line (K562), K562 imatinib-resistant cell line (K562 IR), peripheral blood leukocytes of CML patients in chronic phase (CP), accelerated phase (AP), and blastic phase (BP). MW is obtained using a DNA molecular weight ladder (0.019 to 1.11 k base pair shown). ***b:*** *Sequencing strategy.* The coding gene consists of 6 exons and 3 introns, and an alternative splicing of pre messenger RNA can generate several Bim isoforms [11], [12]. Bim_EL_, Bim_L_ and Bim_S_ are the three main isoforms. Pink arrows represent the E1 and UTR PCR primers for BIM cDNA amplification. E1 is located in the untranslated exon 1 and the UTR is located in the 3' UTR region of the BIM gene. Yellow arrows in exon 3 (green box) are Bim _EL_ specific sequencing primers (forward and reverse). (Exon 3 is excised in Bim_L_ and Bim_S_ ). ***c*** *: Electrophoregrams showing the 3 genotypes for the c465C>T SNP*. Homozygous C/C genotype (upper chart) ; C being the ancestral allele, homozygous T/T genotype (middle chart), and heterozygous C/T genotype (lower chart). Black arrows indicate the nucleotide substitution.
(TIF)
