# Supplementary figures and images for: A Single Nucleotide Polymorphism in cBIM Is Associated with a Slower Achievement of Major Molecular Response in Chronic Myeloid Leukaemia Treated with Imatinib
Source: PLoS One. 2013 Nov 5;8(11):e78582. doi: 10.1371/journal.pone.0078582 (PMC3818406; doi:10.1371/journal.pone.0078582)

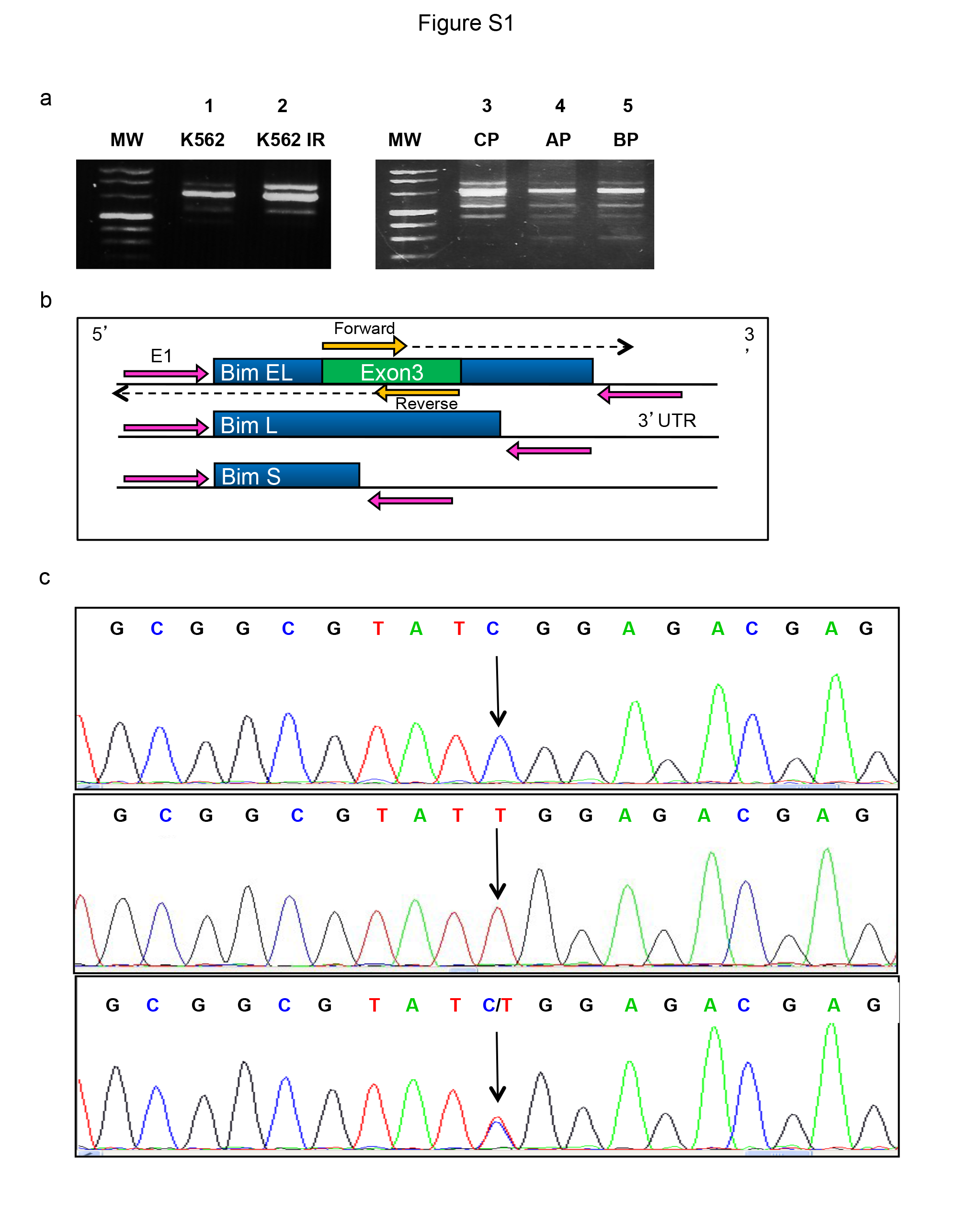

Supplement: Figure S1 — BIMEL cDNA amplification and sequencing. a: Agarose gel electrophoresis of PCR products after BIM cDNA amplification. PCR products after BIM cDNA amplification were analysed by agarose gel electrophoresis. cDNAs were obtained from: K562 imatinib-sensitive cell line (K562), K562 imatinib-resistant cell line (K562 IR), peripheral blood leukocytes of CML patients in chronic phase (CP), accelerated phase (AP), and blastic phase (BP). MW is obtained using a DNA molecular weight ladder (0.019 to 1.11 k base pair shown). b: Sequencing strategy. The coding gene consists of 6 exons and 3 introns, and an alternative splicing of pre messenger RNA can generate several Bim isoforms [11], [12]. BimEL, BimL and BimS are the three main isoforms. Pink arrows represent the E1 and UTR PCR primers for BIM cDNA amplification. E1 is located in the untranslated exon 1 and the UTR is located in the 3' UTR region of the BIM gene. Yellow arrows in exon 3 (green box) are Bim EL specific sequencing primers (forward and reverse). (Exon 3 is excised in BimL and BimS ). c : Electrophoregrams showing the 3 genotypes for the c465C>T SNP. Homozygous C/C genotype (upper chart) ; C being the ancestral allele, homozygous T/T genotype (middle chart), and heterozygous C/T genotype (lower chart). Black arrows indicate the nucleotide substitution. (TIF) [file pone.0078582.s001.tif]
